# Supplementary figures and images for: A revised understanding of Tribolium morphogenesis further reconciles short and long germ development
Source: PLoS Biol. 2018 Jul 3;16(7):e2005093. doi: 10.1371/journal.pbio.2005093 (PMC6047830; doi:10.1371/journal.pbio.2005093)

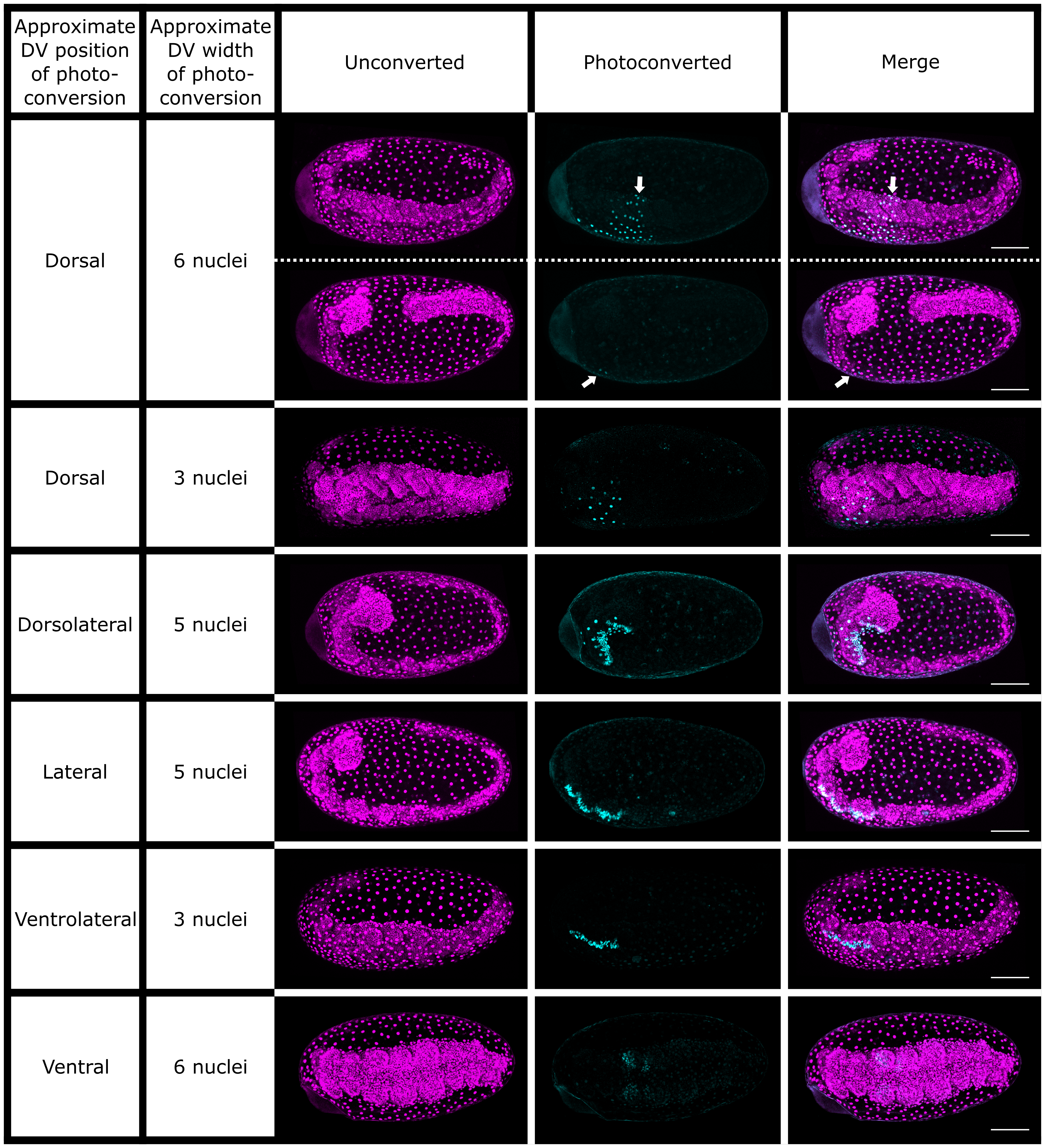

Supplement: S1 Fig — NLS-tdEos-labelled extended germband stage Tribolium embryos in which a patch of blastoderm nuclei was photoconverted at 50% egg length (from the posterior pole) at different DV positions. The approximate DV position of the patch and the approximate DV width of the clone (in terms of nuclei number) are shown. The dorsal labelled embryo is shown from both sides to demonstrate the photoconverted nuclei cover the full DV extent of the amnion (arrows). Unconverted protein is shown in magenta; converted protein is shown in cyan. Images are maximum intensity projections of one egg hemisphere. All eggs are oriented with the anterior to the left and ventral to the bottom. Scale bars are 100 μm. DV, dorsoventral; NLS-tdEos, nuclear localisation signal-tandem Eos. (TIF) [file pbio.2005093.s001.tif]

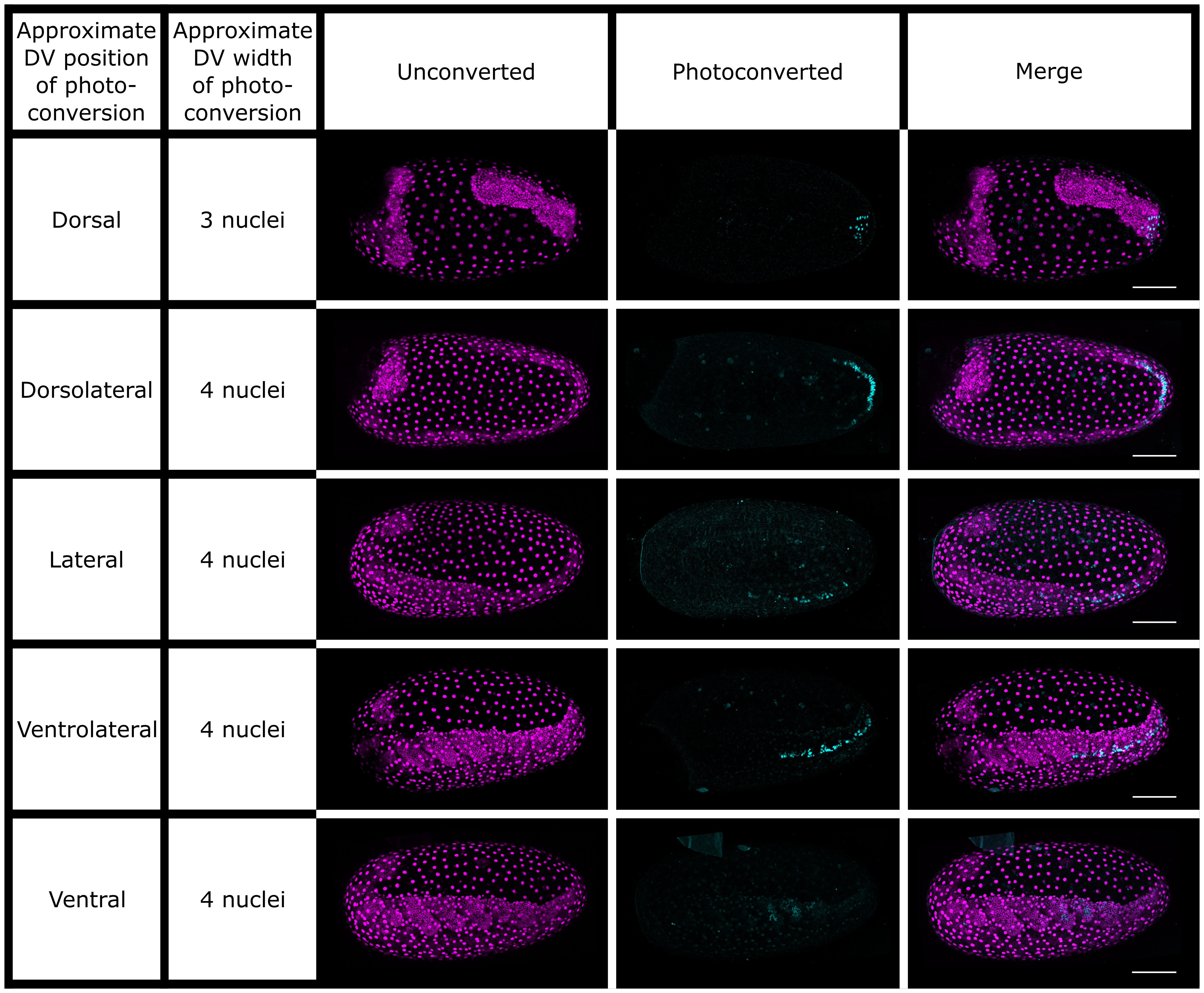

Supplement: S2 Fig — NLS-tdEos-labelled extended germband stage Tribolium embryos in which a patch of blastoderm nuclei were photoconverted at 25% egg length (from the posterior pole) at different DV positions. The approximate DV position of the patch and the approximate DV width of the clone (in terms of nuclei number) are shown. Unconverted protein is shown in magenta; converted protein is shown in cyan. Images are maximum intensity projections of one egg hemisphere. All eggs are oriented with the anterior to the left and ventral to the bottom. Scale bars are 100 μm. DV, dorsoventral; NLS-tdEos, nuclear localisation signal-tandem Eos. (TIF) [file pbio.2005093.s002.tif]

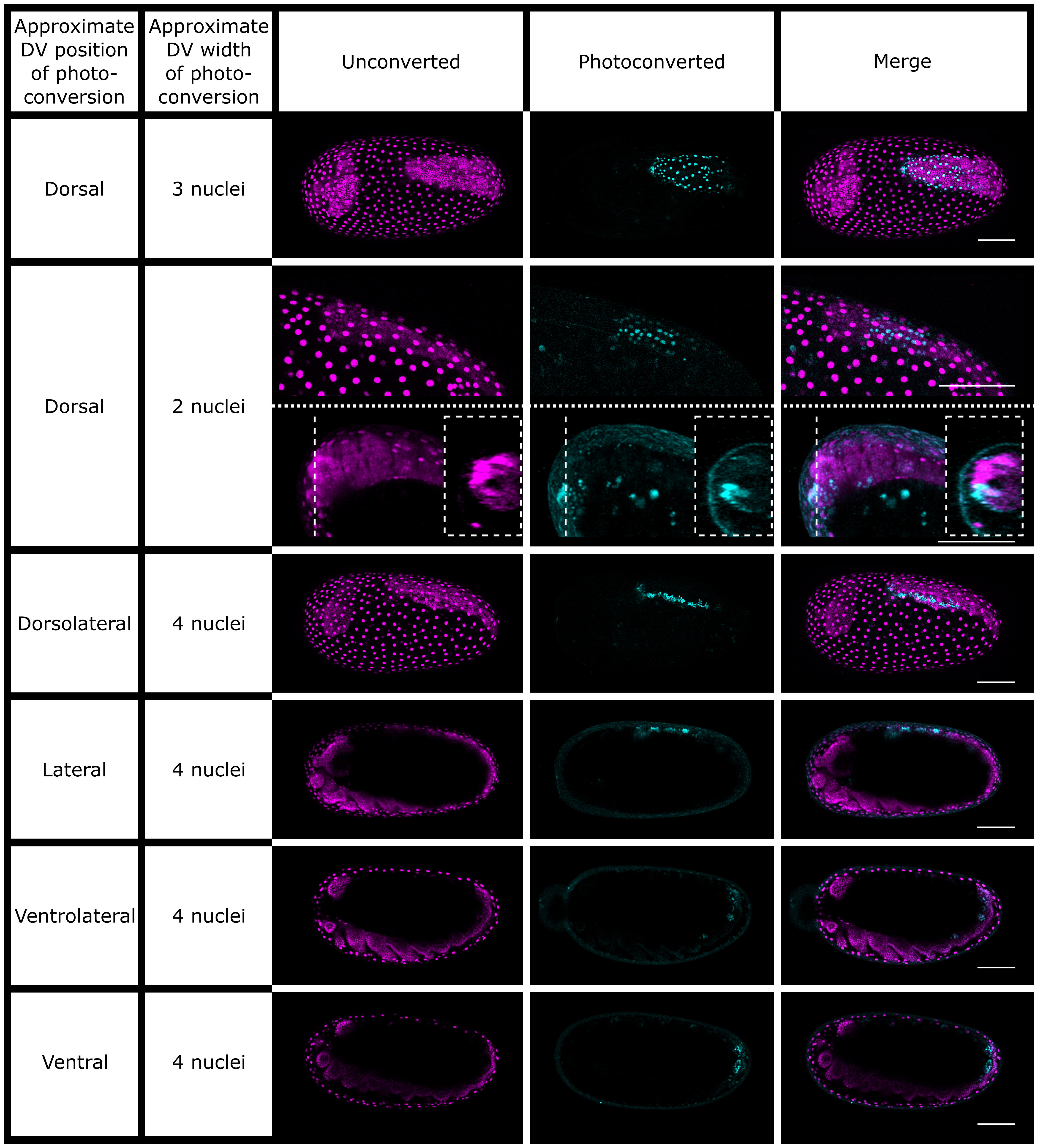

Supplement: S3 Fig — NLS-tdEos-labelled extended germband stage Tribolium embryos in which a patch of blastoderm nuclei were photoconverted near the posterior pole at different DV positions. The approximate DV position of the patch and the approximate DV width of the clone (in terms of nuclei number) are shown. The second dorsally labelled embryo is shown at high magnification at two time points and with a transverse section (at the position of the dashed green line) to show the movement of tissue from the dorsal epithelium into the hindgut. Unconverted protein is shown in magenta; converted protein is shown in cyan. Images are maximum intensity projections of one egg hemisphere except for the bottom three embryos, which are shown as maximum intensity projects through the germband in order to better show the labelled nuclei. All eggs are oriented with the anterior to the left and ventral to the bottom except for the second time point of the second dorsal view, which is shown with the posterior of the germband to the left. Scale bars are 100 μm. DV, dorsoventral; NLS-tdEos, nuclear localisation signal-tandem Eos. (TIF) [file pbio.2005093.s003.tif]

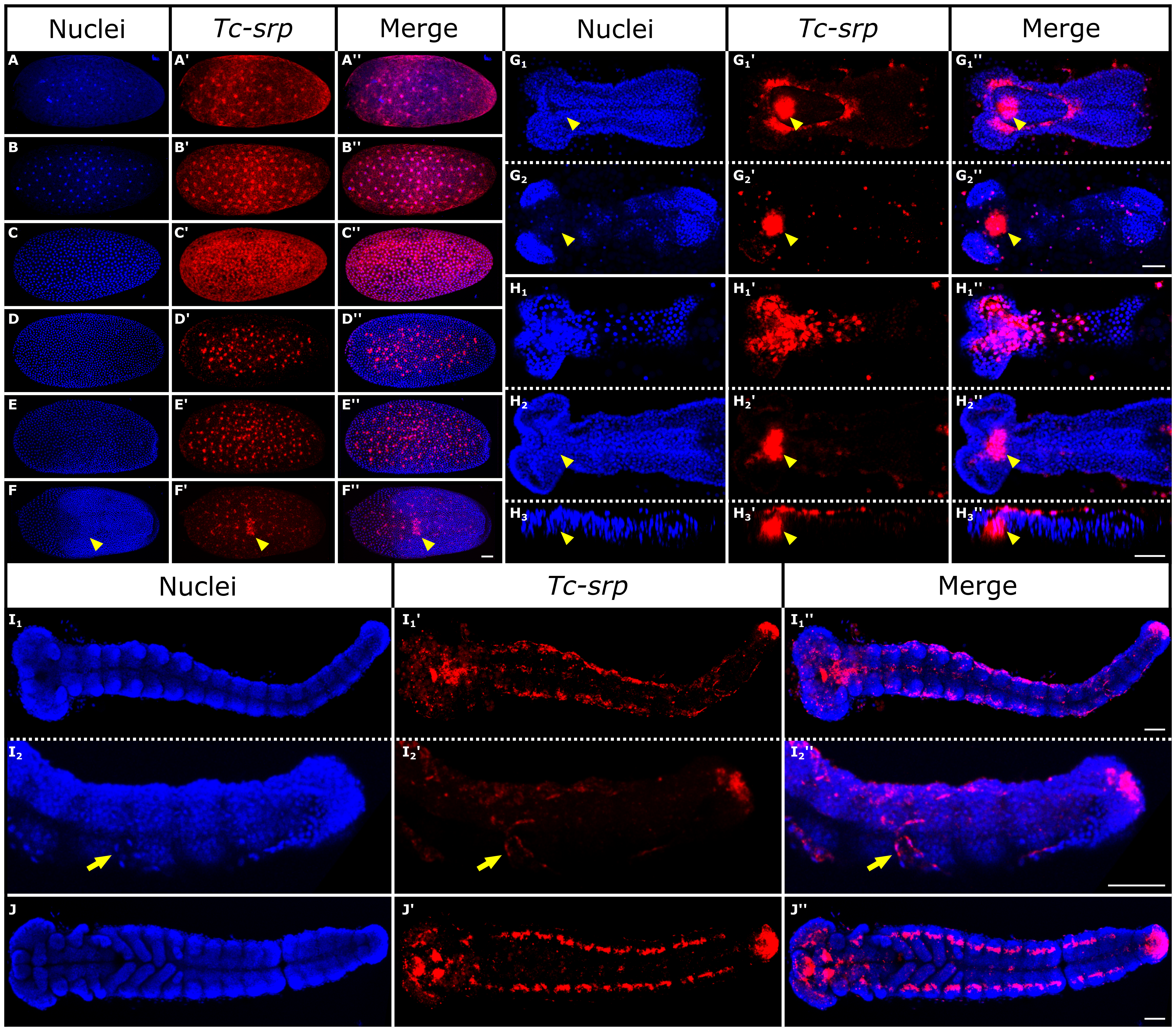

Supplement: S4 Fig — (A-F) whole mount and (G-J) flat-mount Tribolium embryos from the pre-blastoderm to the retracting germband stage stained for Tc-srp mRNA (red) and nuclei (DAPI, blue). (G1) and (G2) show the same embryo imaged from both sides. (H1) and (H2) show projections from the dorsal epithelium (H1) and the ventral epithelium (H2) of the same embryo. Tc-srp mRNA is maternally provided (A), and expression is ubiquitous until the late blastoderm stage (B-C), when expression clears from the blastoderm but persists in the yolk nuclei (scattered spots in [D-E]). During embryo condensation, de novo expression arises in a patch of blastoderm cells at the anterior medial region (arrowhead in F). This patch of Tc-srp-expressing cells invaginates as part of the ventral furrow and becomes located beneath the ectoderm (arrowhead in G1–H3). This expression domain is likely homologous to the anterior ventral expression domain in Drosophila that marks the prohemocytes. During serosa window closure, expression appears in a ring of dorsal epithelium cells (G1). After serosa window closure, expression persists in the dorsal epithelium (H1) and (H3). Unlike Drosophila, there is no expression domain at the posterior of the blastoderm (E) or the early germband (G2). After germband elongation, a de novo expression domain appears at the posteriormost point of the embryo (I1). Given the location of this domain at the base of the forming hindgut, this is likely the posterior endoderm primordium. Expression can also be seen in a patch of amnion that has remained attached to the germband (arrow in I2), but most of the rest of the amnion has been lost. Several other regions of expression can be seen, including in the presumptive fat body (the segmental domains running down the body), in presumptive hemocyte clusters (the two side-by-side domains in the anterior), and in an anterior domain that may mark the anterior endoderm primordium. Expression also persists in the yolk nuclei (visible in the remainin [file pbio.2005093.s004.tif]

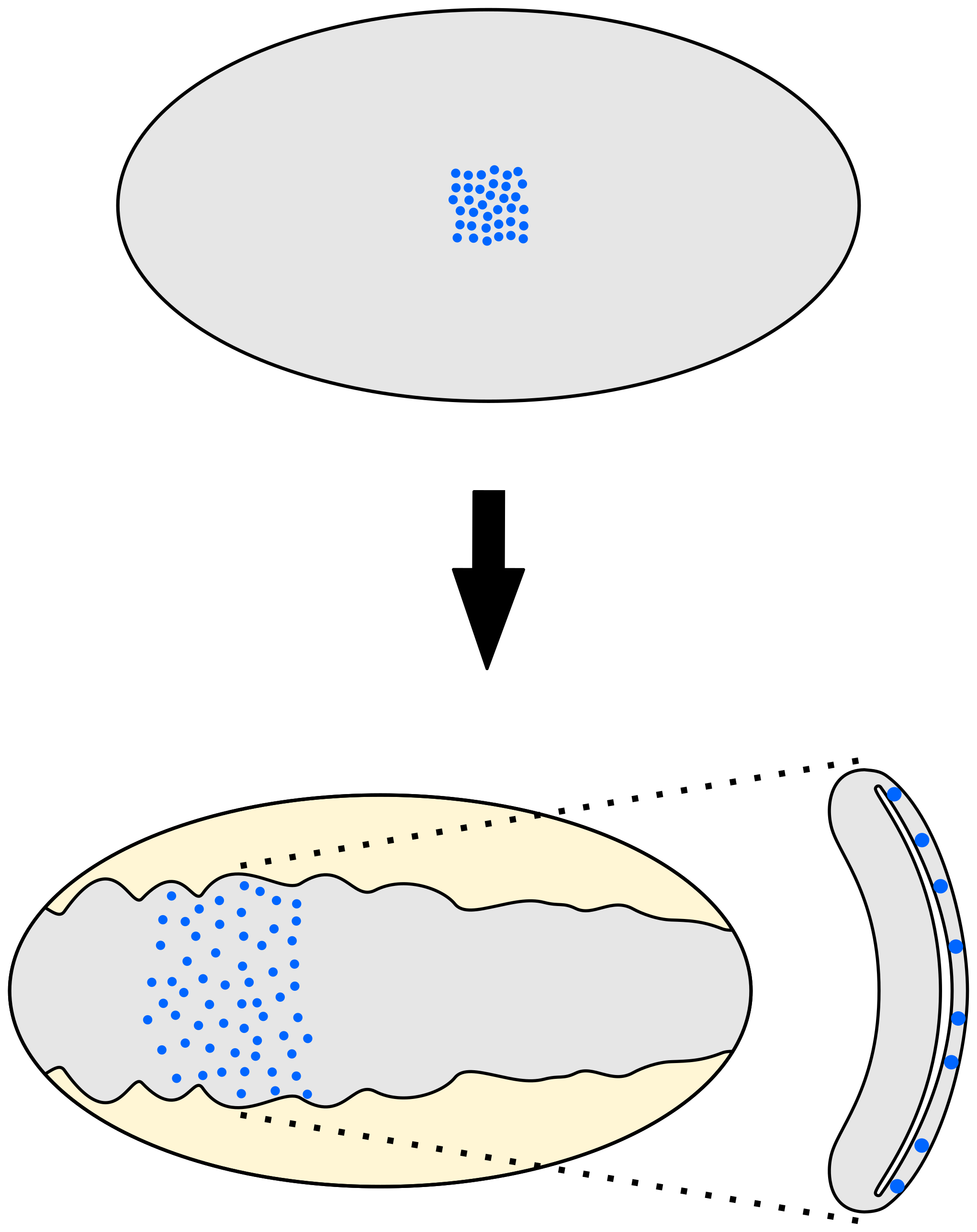

Supplement: S5 Fig — A patch of nuclei (of known dimensions) was photoconverted at the blastoderm stage, then the same embryos were examined at the end of germband extension. In embryos in which all photoconverted nuclei were located in the amnion and these nuclei spanned the entire DV width of the amnion (as shown here), the number of nuclei initially photoconverted was used to determine the DV width of the blastoderm domain giving rise to the amnion. Note that the precise number and distribution of nuclei shown here were arbitrarily chosen. Blue shows photoconverted nuclei; yellow shows the yolk. The serosa is omitted from the bottom panels. DV, dorsoventral. (TIF) [file pbio.2005093.s005.tif]

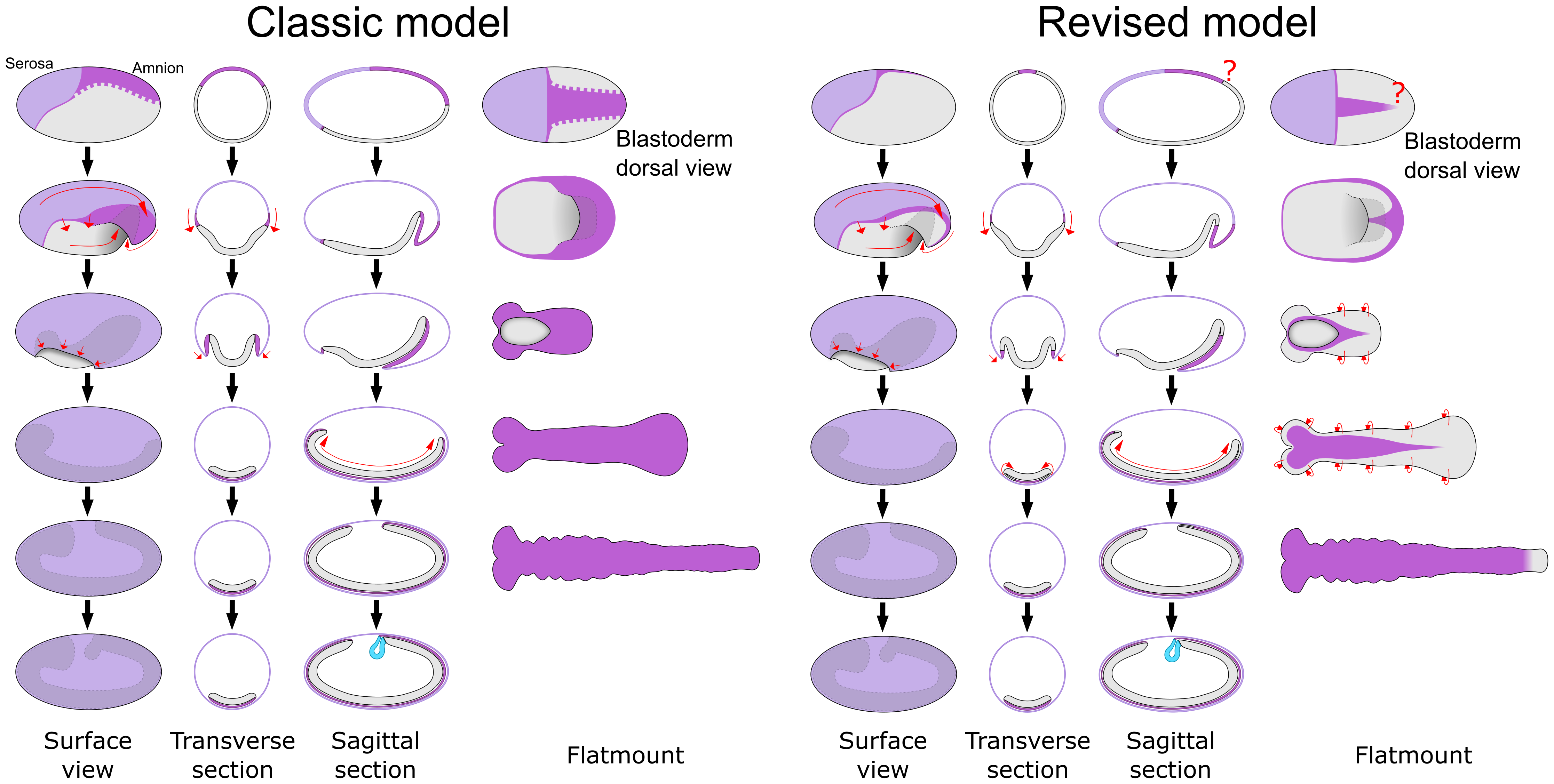

Supplement: S6 Fig — Schematics drawn as in Fig 1 to show the classic and revised fate maps and germband models based on the results of this manuscript. The schematics of the flat-mounted germbands are drawn with the focus on the dorsal epithelium. See text for additional details. (TIF) [file pbio.2005093.s006.tif]

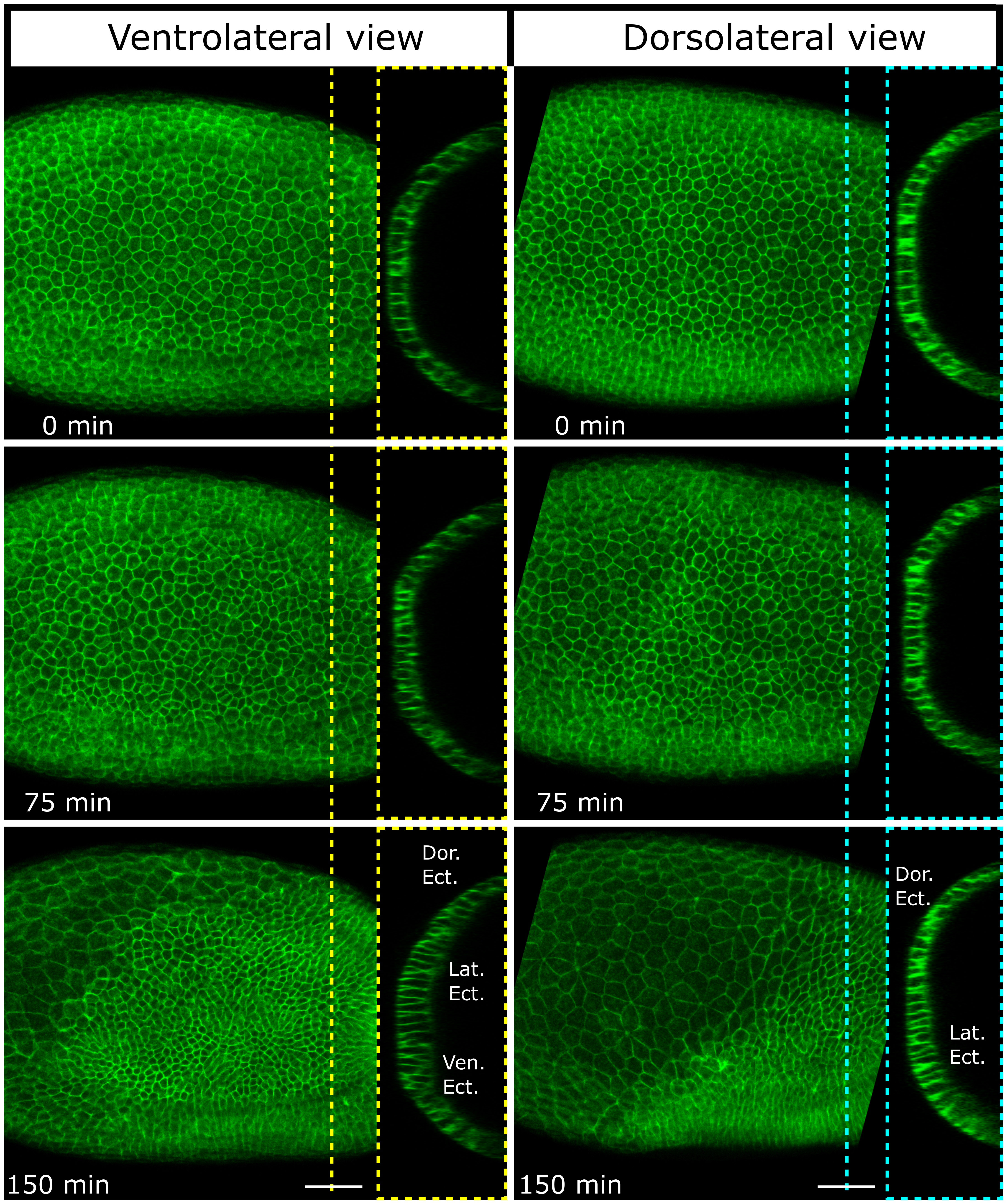

Supplement: S7 Fig — Stills from timelapses of two Tribolium embryos transiently expressing GAP43YFP to label membranes. The second panel of each time point shows optical transverse sections at the position of the dashed line in the related panel. Ventral and lateral ectoderm becomes columnar, while dorsal ectoderm becomes flattened. The non-columnar cells at the bottom of the left hand embryo are likely the presumptive mesoderm. The first frame of the timelapses was defined as time point 0. Both embryos are oriented with the anterior to the left and ventral to the bottom. Scale bars are 100 μm. Dor, Dorsal; Ect, Ectoderm; GAP43YFP, GAP43-yellow fluorescent protein Lat, Lateral; Ven, Ventral. (TIF) [file pbio.2005093.s007.tif]
