# Supplementary material for: A revised understanding of Tribolium morphogenesis further reconciles short and long germ development
Source: PLoS Biol. 2018 Jul 3;16(7):e2005093. doi: 10.1371/journal.pbio.2005093 (PMC6047830; doi:10.1371/journal.pbio.2005093)
Supplement: S1 Text — (DOCX) [file pbio.2005093.s015.docx]

**A revised understanding of the short germ embryo**

**A possible mechanism of BMP function in the germband**

In *Drosophila*, the BMP pathway is activated by the extracellular ligand *decapentaplegic* (*dpp*), which accumulates on the dorsal side by a combination of local *dpp* expression and ventral-to-dorsal protein shuttling involving the BMP repressor *short gastrulation* (*sog*) (reviewed in [1]). The same mechanism is thought to occur in the *Tribolium* blastoderm, where the *Tribolium* ortholog of *sog* is expressed in a broad ventral domain and BMP activity is restricted to the dorsal side [2]. In the germband, the same domains of *sog* expression and BMP activity exist [2,3]. As such, the cells in the dorsal epithelium could be exposed to high levels of BMP activity, while cells in the ventral epithelium could be exposed to no BMP activity (due to extracellular *sog*). However, cells move from the dorsal epithelium to the ventral epithelium, and therefore, they would move from the ‘activator region’ (extracellular *dpp*) to the ‘repressor region’ (extracellular *sog*) in this model. This movement from one region to another correlates with the change in cell shape from flattened to columnar as they enter the ventral epithelium, exactly as expected if BMP activity maintains cells in a flattened state. If this system does indeed function as hypothesised here, it would confer some degree of developmental robustness, as BMP activity would both effect dorsal-to-ventral tissue flow, and be affected by dorsal-to-ventral tissue flow.

Regardless of how exactly the system works, one real effect of the tissue flow and BMP activity dynamics is that the number of cells with active BMP signalling decreases over time. This means that although the visible width of the active BMP domain appears static, the width in terms of cell number decreases. As such, the initially broad domain of BMP activity present at the late blastoderm stage narrows during GBE, just as occurs in *Drosophila* [4]. During later maturation of germband tissue, BMP activity does become visible in the dorsal margins of the germband [2], and this also occurs in *Drosophila* [4].

**References**

1. Moussian B, Roth S. Dorsoventral axis formation in the Drosophila embryo--shaping and transducing a morphogen gradient. Curr Biol. 2005;15: R887-99. doi:10.1016/j.cub.2005.10.026

2. van der Zee M, Stockhammer O, von Levetzow C, Nunes da Fonseca R, Roth S. Sog/Chordin is required for ventral-to-dorsal Dpp/BMP transport and head formation in a short germ insect. Proc Natl Acad Sci U S A. 2006;103: 16307–12. doi:10.1073/pnas.0605154103

3. Schwirz J. Systematic reverse genetic screen to identify novel genes required for anterior patterning of the red flour beetle Tribolium castaneum. Georg-August-Universitaet Goettingen. 2014.

4. Dorfman R, Shilo BZ. Biphasic activation of the BMP pathway patterns the Drosophila embryonic dorsal region. Development. 2001;128: 965 LP-972. Available: http://dev.biologists.org/content/128/6/965.abstract
