# Supplementary material for: A revised understanding of Tribolium morphogenesis further reconciles short and long germ development
Source: PLoS Biol. 2018 Jul 3;16(7):e2005093. doi: 10.1371/journal.pbio.2005093 (PMC6047830; doi:10.1371/journal.pbio.2005093)
Supplement: S2 Text — (DOCX) [file pbio.2005093.s016.docx]

**Cellular and molecular causes of tissue flow**

**Cell intercalation**

During *Tribolium* embryo condensation, ectodermal cells undergo mediolateral cell intercalation, which in turn causes the narrowing and extension (convergent extension) of the embryonic tissue [1]. This cell intercalation continues in the posterior of the embryo during condensation and throughout GBE, and is a known driver of both events [2]. Our previous model of this process was that mediolateral cell intercalation only took place in the ventral epithelium (Fig 1). However, the genes driving intercalation (*Tc-Toll7* and *Tc-Toll10*) are expressed in rings around the entire germband epithelium [3], and therefore, it is likely that cells of the dorsal epithelium are also undergoing mediolateral intercalation. This conclusion is supported by the fact that dorsalised *Tribolium* germbands (i.e. that lack ventral tissue) undergo extensive GBE (discussed below).

**Tissue specific cell shape changes**

Tissue specific cell shape changes take place at both the condensation and germband stages. In contrast to the columnar epithelium of the *Drosophila* blastoderm [4], the *Tribolium* blastoderm initially consists of an epithelium of cuboidal cells (as is also true of most insects; [5]). Then, as condensation takes place, cells of the ventrolateral and lateral ectoderm undergo a cuboidal-to-columnar transition. This change in shape causes ventrolateral and lateral cells to become both taller and thinner (Supplemental Fig 7), meaning that the apical and basal surface areas decrease. The tissue level consequence of this change is an overall decrease of the surface area of this region of the ectoderm. At the same time, the dorsal/dorsolateral ectoderm cells become shorter and wider (Supplemental Fig 7), thereby increasing the overall surface area of this region of the ectoderm. These tissue specific cell shape changes are likely a key cause of the germband condensing specifically on the ventral side of the egg [6]. Later, during GBE, the same tissue specific cell shape changes are also occurring. Cells at the lateral edges of the germband are columnar [7], and therefore, when cells move from the dorsal epithelium to the ventral epithelium they must become columnar. Meanwhile, cells that remain in the dorsal epithelium become squamous (Fig 4). As such, ventrolateral/lateral ectoderm becomes columnar, while dorsal ectoderm becomes squamous. It is important to note, however, that dorsal/dorsolateral cells do undergo a brief columnar phase as the germband proper forms (at the serosa window stage) [7,8]. As such, thinning of dorsal cells during condensation and GBE is not a temporally continuous process (in contrast to the mediolateral cell intercalation described earlier).

In contrast to cell intercalation, almost nothing is known about the molecular control of the cell shape changes. It is clear, however, that at the highest level this event must be controlled by DV axis patterning components, as cells differentially change shape along the DV axis. In line with this, BMP signalling appears to be involved in flattening of dorsal cells during both condensation and GBE. At the blastoderm stage, BMP signalling becomes active in a broad peak on the dorsal side of the embryonic rudiment (as shown by pMAD staining) [9]. This domain disappears during the second half of condensation [10], but reappears shortly afterwards in the dorsal epithelium (see figures 4-16, 4-17, and 4-30 of [11]). This temporal pattern (active during the beginning of condensation, inactive during serosa window closure, active during GBE) correlates with the changes in the shapes of these cells (flattened during the beginning of condensation, columnar during serosa window closure, then flattened during GBE), exactly as we would expect if the cell shape changes are controlled by BMP signalling. Furthermore, disruption of BMP signalling specifically at the GB stage causes defects in GBE [10], possibly due to disruption of the dorsal tissue flattening.

The strongest support for the cell shape changes being controlled by DV axis patterning comes from the phenotypes caused by the complete disruption of DV axis specification components. Disruption of either DV specification genes causes condensation to occur in a more radially symmetric manner than in wildtype [9,12]. The resulting germbands are shaped like tubes, rather than the usual flattened cylinder, with uniform cell shapes around the entire ectoderm. In addition, the germband ‘tubes’ are substantially narrower and longer than wildtype germbands, and this aspect of the phenotype was not previously understood. With the new model of *Tribolium* development, it is a simple matter to explain the aetiology of this phenotype. Based on the model, flattening of dorsal ectoderm and simultaneous increase in height of ventral ectoderm is one event that causes (or perhaps facilitates) dorsal-to-ventral cell flow. Secondly, mediolateral cell intercalation causes the ectoderm as a whole to converge along the DV axis and extend along the AP axis. In the absence of any DV asymmetry in cell shape, the intercalation driven tissue convergence would occur uniformly around DV axis of the ectoderm (instead of being directed towards the ventral midline). As the ectoderm is continuous along the DV axis (forming a tube), the uniform tissue convergence would cause the overall narrowing of the tube (as occurs in several other embryonic tube structures; [13]). The reason that the germband extends more than usual is because the lack of DV differences in tissue properties would allow all cells around the tube to intercalate equally.

**References**

1. Benton MA, Akam M, Pavlopoulos A. Cell and tissue dynamics during Tribolium embryogenesis revealed by versatile fluorescence labeling approaches. Development. 2013;140: 3210–3220. doi:10.1242/dev.096271

2. Nakamoto A, Hester SD, Constantinou SJ, Blaine WG, Tewksbury AB, Matei MT, et al. Changing cell behaviours during beetle embryogenesis correlates with slowing of segmentation. Nat Commun. Nature Publishing Group; 2015;6: 6635. doi:10.1038/ncomms7635

3. Benton MA, Pechmann M, Frey N, Stappert D, Conrads KHKH, Chen Y-TY-T, et al. Toll Genes Have an Ancestral Role in Axis Elongation. Curr Biol. Elsevier Ltd; 2016;26: 1609–1615. doi:10.1016/j.cub.2016.04.055

4. Turner FR, Mahowald AP. Scanning electron microscopy of Drosophila embryogenesis I the structure of the egg envelopes and the formation of the cellular blastoderm. Dev Biol. 1976;50: 95–108. doi:10.1016/0012-1606(76)90070-1

5. van der Zee M, Benton MA, Vazquez-Faci T, Lamers GEM, Jacobs CGC, Rabouille C. Innexin7a forms junctions that stabilize the basal membrane during cellularization of the blastoderm in Tribolium castaneum. Development. 2015;142: 2173–2183. doi:10.1242/dev.097113

6. Benton MA, Pavlopoulos A. Tribolium embryo morphogenesis: May the force be with you. Bioarchitecture. 2014;4: 16–21. doi:10.4161/bioa.27815

7. Handel K, Basal A, Fan X, Roth S. Tribolium castaneum twist: gastrulation and mesoderm formation in a short-germ beetle. Dev Genes Evol. 2005;215: 13–31.

8. Handel K, Grünfelder CG, Roth S, Sander K. Tribolium embryogenesis: a SEM study of cell shapes and movements from blastoderm to serosal closure. Dev Genes. 2000;

9. van der Zee M, Stockhammer O, von Levetzow C, Nunes da Fonseca R, Roth S. Sog/Chordin is required for ventral-to-dorsal Dpp/BMP transport and head formation in a short germ insect. Proc Natl Acad Sci U S A. 2006;103: 16307–12. doi:10.1073/pnas.0605154103

10. Horn T, Panfilio KA. Novel functions for Dorsocross in epithelial morphogenesis in the beetle Tribolium castaneum. Development. 2016;143: 3002–3011. doi:10.1242/dev.133280

11. Schwirz J. Systematic reverse genetic screen to identify novel genes required for anterior patterning of the red flour beetle Tribolium castaneum. Georg-August-Universitaet Goettingen. 2014.

12. Nunes da Fonseca R, von Levetzow C, Kalscheuer P, Basal A, van der Zee M, Roth S. Self-Regulatory Circuits in Dorsoventral Axis Formation of the Short-Germ Beetle Tribolium castaneum. Dev Cell. 2008;14: 605–615. doi:https://doi.org/10.1016/j.devcel.2008.02.011

13. Walck-Shannon E, Hardin J. Cell intercalation from top to bottom. Nat Rev Mol Cell Biol. Nature Publishing Group; 2013;15: 34–48. doi:10.1038/nrm3723
