# Supplementary material for: A revised understanding of Tribolium morphogenesis further reconciles short and long germ development
Source: PLoS Biol. 2018 Jul 3;16(7):e2005093. doi: 10.1371/journal.pbio.2005093 (PMC6047830; doi:10.1371/journal.pbio.2005093)
Supplement: S3 Text — (DOCX) [file pbio.2005093.s017.docx]

**Reconciling long and short germ development**

As described earlier, the *Tribolium* blastoderm ectoderm initially consists of cuboidal cells that then become columnar as they develop. In contrast, cellularisation in *Drosophila* directly produces columnar cells [1]. This derived mode of cellularisation is likely associated with carrying out extensive tissue patterning early and rapidly. Despite this change, differences in ectodermal cells along the DV axis are clearly visible in *Drosophila*. Initially, during gastrulation and the beginning of GBE, dorsal cells are shorter than the rest of the ectoderm [2] and display quantitative differences in apical actomyosin distribution and apical shape changes [3]. Later, during GBE, dorsal cells shorten drastically and undergo “rotary cell elongation” to yield mature, stretched out amnioserosa cells [4]. Rather than purely being the result of mechanical causes, these tissue specific cell shape changes are genetically controlled by DV specification components [2–4], as are those in *Tribolium*.

**References**

1. Turner FR, Mahowald AP. Scanning electron microscopy of Drosophila embryogenesis I the structure of the egg envelopes and the formation of the cellular blastoderm. Dev Biol. 1976;50: 95–108. doi:10.1016/0012-1606(76)90070-1

2. Leptin M, Grunewald B. Cell shape changes during gastrulation in Drosophila. Development. 1990;110: 73 LP-84. Available: http://dev.biologists.org/content/110/1/73.abstract

3. Rauzi M, Krzic U, Saunders TE, Krajnc M, Ziherl P, Hufnagel L, et al. Embryo-scale tissue mechanics during Drosophila gastrulation movements. Nat Commun. The Author(s); 2015;6: 8677. Available: http://dx.doi.org/10.1038/ncomms9677

4. Pope KL, Harris TJC. Control of cell flattening and junctional remodeling during squamous epithelial morphogenesis in Drosophila. Development. 2008;135: 2227–38. doi:10.1242/dev.019802
